# Foraging for the future: traditional culinary uses of wild plants in the Western Himalayas–Kashmir Valley (India)

**DOI:** 10.1186/s13002-024-00707-7

**Published:** 2024-07-13

**Authors:** Musheerul Hassan, Tawseef Ahmad Mir, Muatasim Jan, Muhammad Shoaib Amjad, Muhammad Abdul Aziz, Andrea Pieroni, Ivana Vitasović-Kosić, Rainer W. Bussmann

**Affiliations:** 1 Department of Ethnobotany, Institute of Botany, Ilia State University, 0105 Tbilisi, Georgia; 2 Alpine Institute of Management and Technology, Dehradun, Uttarakhand 248007 India; 3 Centre of Research for Ethnobotany, Government Model Science College, Jiwaji University, Gwalior, 474009 India; 4 Faculty of Botany and Forestry, BFIT Group of Institutions, Dehradun, Uttarakhand 248007 India; 5 IUCN, Gland, Switzerland; 6 Department of Botany, Women University of Azad Jammu and Kashmir Bagh, Bagh, 12500 Pakistan; 7 Birmingham Institute of Forest Research, University of Birmingham, Birmingham, B15 2TT UK; 8 Department of Environmental Sciences, Informatics, and Statistics, Ca’ Foscari University of Venice, Via Torino 155, 30172 Venezia, Italy; 9 University of Gastronomic Sciences, Piazza Vittorio Emanuele II 9, 12042 Pollenzo, Bra, Italy; 10 Department of Medical Analysis, Tishk International University, Erbil, Kurdistan 44001 Iraq; 11 Division of Horticulture and Landscape Architecture, Department of Agricultural Botany, Faculty of Agriculture, University of Zagreb, Svetošimunska cesta 25, 10000 Zagreb, Croatia; 12 Department of Botany, State Museum of Natural History, 76133 Karlsruhe, Germany

**Keywords:** Wild vegetable, Traditional knowledge, Ethno-gastronomy, Kashmir Valley, Edible fungi

## Abstract

**Background:**

In the intricate tapestry of food security, wild food species stand as pillars, nourishing millions in low-income communities, and reflecting the resilience and adaptability of human societies. Their significance extends beyond mere sustenance, intertwining with cultural traditions and local knowledge systems, underscoring the importance of preserving biodiversity and traditional practices for sustainable livelihoods.

**Methods:**

The present study, conducted between February 2022 and August 2023 along the Line of Control in India’s Kashmir Valley, employed a rigorous data collection encompassing semi-structured interviews, focus group discussions, and specific field observations facilitated through a snowball sampling technique.

**Results and discussion:**

The comprehensive inventory includes 108 edible plant and fungal species from 48 taxonomic families, with Rosaceae (*N* = 11) standing out. Young and soft leaves (N = 60) are an important component of various culinary preparations, with vegetables (*N* = 65) being the main use, followed by fruits (*N* = 19). This use is seasonal, with collection peaks in March–April and June–August (*N* = 12). The study also highlights the importance of use value (UV), with *Portulaca oleracea* standing out as the plant taxon (UV = 0.61), while *Asyneuma thomsoni* has the lowest use value (UV = 0.15). Many species such as *Senecio chrysanthemoides, Asperugo procumbens, Asyneuma thomsoni,* and *Potentilla nepalensis* were classified as new for gastronomic use. Furthermore, the study underlines the great cultural importance of mushrooms such as *Morchella esculenta* and *Geopora arenicola* in influencing social hierarchies within the community. However, the transmission of traditional knowledge across generations is declining in the region. At the same time, the conservation of endangered plant species on the IUCN Red List, such as *Trillium govanianum, Taxus wallichiana, Saussurea costus,* and *Podophyllum hexandrum*, requires immediate attention.

**Conclusion:**

Conservation measures should be prioritized, and proactive remedial action is needed. Further research into the nutritional value of these edible species could pave the way for their commercial cultivation, which would mean potential economic growth for local communities, make an important contribution to food security in the area under study, and contribute to scientific progress.

## Background

Wild foods, encompassing plants and fungi that flourish in natural environments without human intervention, constitute a fundamental resource harvested by numerous rural communities globally [1]. The practice of gathering wild foods and incorporating them into daily diets has become widespread, significantly enhancing the nutritional status [2, 3]. This practice serves the dual purpose of reducing reliance on commercial food sources and bolstering food security. The indigenous wisdom underlying these practices is invaluable, highlighting its practical benefits. Wild edible species are crucial in sustaining millions of people, particularly in rural and impoverished regions [4]. The integration of indigenous wisdom and ethno-scientific methods into contemporary conservation and sustainable resource management practices is increasingly vital [5]. Such integration is crucial for constructing resilient and sustainable food systems. This approach aligns with Article 8(j) of the Convention on Biological Diversity (CBD), which underscores the importance of traditional knowledge (TK) in the development of sustainable food systems in specific regions [5, 6]. Furthermore, biocultural refugia serve as repositories of traditional knowledge, preserving the essence of conventional food systems and historically playing a central role in safeguarding communities during famines [7].

Understanding the age-old practice of traditional plant foraging, deeply embedded in local customs, is essential as it catalyzes the emergence of new gastronomic identities while supporting the sustainability of isolated indigenous food systems [8]. Reviving and examining the biocultural culinary heritage that underpins the development of indigenous gastronomy on a global scale reveals the indispensable role of traditional knowledge (TK). This knowledge offers a wealth of ancient ingredients, forgotten plants, specific collection times for particular species, and cultural significance, which, when harnessed, support the fight against food insecurity [9].

However, significant conservation issues are associated with the harvesting of wild foods. Overharvesting, climate change, and urbanization pose substantial threats to these resources. Unsustainable practices can lead to the depletion of wild species, undermining ecological balance and the availability of these critical food sources for future generations [10, 11]. Indigenous communities are custodians of vanishing botanical knowledge and ancient ecological narratives. The global trend of urbanization is disrupting their way of life, impeding the intergenerational transmission of knowledge, and potentially leading to the erosion of traditional knowledge. [12, 13]. In this regard, the urgency to preserve this reservoir of knowledge is unmistakable and necessitates its careful integration into sustainable food and health paradigms, as strongly advocated by Aziz et al. [14]. Developing a concrete plan to protect this invaluable treasure from the relentless passage of time and the unstoppable force of modernization requires intensive participation in extensive ethnobotanical field studies. As part of our endeavor, the present study aims to document the traditional knowledge of wild food species among the people living in Kashmir. The primary objectives of our scientific inquiry are twofold: firstly, to meticulously document the diverse edible wild food species prevalent among the populace residing along the Line of Control within the Kashmir Valley of India, and secondly, to comprehensively record essential aspects of the primary gastronomic uses, utilized plant parts, temporal patterns of collection, cultural importance, intergenerational dissemination of traditional culinary knowledge, and conservation statuses associated with these documented species.

## Materials and methods

### Study area

**Fig. 1:**
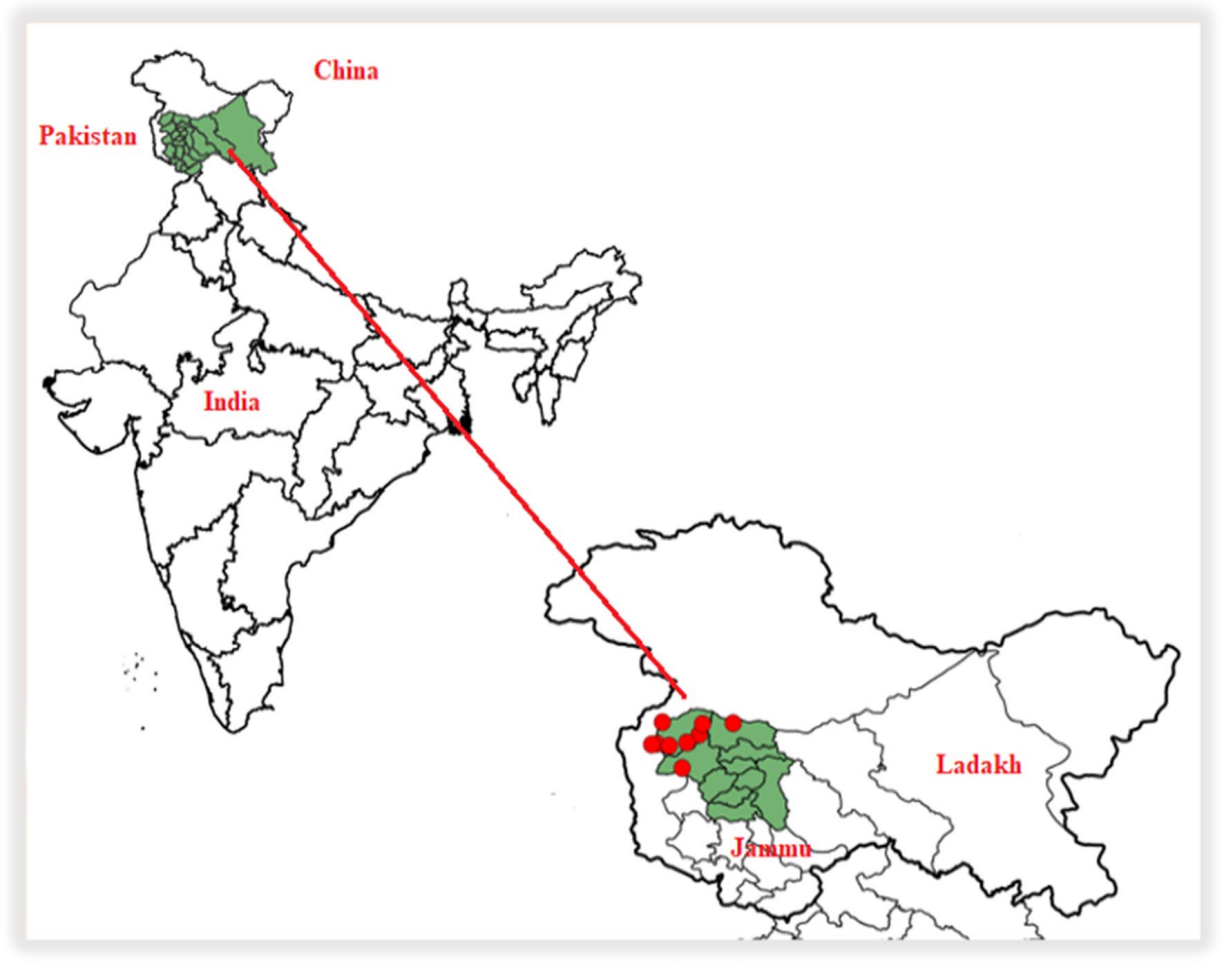
Map of the study area Kashmir Valley (India)

The strategically important Kashmir Valley is located in the northernmost part of India (Fig. 1) and borders China to the northeast, which includes the autonomous Uyghur region of Xinjiang and the autonomous region of Tibet. In the west and northwest, it borders Pakistan, which is demarcated by the Line of Control (LoC) [15]. In addition, the valley is surrounded to the south by other Indian states such as Himachal Pradesh and Punjab.

The region covers an area of around 15,948 square kilometers and has a diverse demography, with Muslims forming the majority (67%) along with Hindus, Sikhs, and Buddhists. Known for its temperate climate and ecological richness, the Kashmir Valley has a mosaic of forest types ranging from the humid temperate zone of the Himalayas to sub-alpine forests. The intricate geopolitical dynamics, cultural heritage, and natural beauty make Kashmir a significant and complex part of the Union Territory. Agriculture is the cornerstone of the Kashmir Valley [16] and is closely associated with various related services. Outside agriculture, the population is engaged in various activities, some in trade, others as day laborers, semi-skilled artisans, and shepherds, all of whom make a unique contribution to the vibrant picture of this land. According to the anthropological survey of India under the People of India project, there are 111 ethnic groups living in erstwhile Jammu and Kashmir [17].

### Data collection

The present study was conducted through field interviews that took place from February 2022 to August 2023 in the Kashmir Valley. A total of 97 informants, including 52% female and 48% male participants aged between 20 and 75 years from *N* = 9 different villages (Table 1), were selected using the snowball method. Data collection included semi-structured interviews, focus group discussions, and field observations following the well-established methods of Manzoor et al. [15] and Mir et al. [18]. Key information included edible wild plant species, their local nomenclature, growth habits, parts used, collection times, market value, medicinal properties, and culinary uses (e.g., vegetables or fruits). The questionnaires were filled in Urdu and Kashmiri and facilitated by pictures and plant specimens collected during the survey, which helped in the identification of the specimens. Where necessary, individual interviews were conducted to supplement the responses to the questionnaires. The study strictly adhered to the ethical guidelines of the International Society for Ethnobiology (https://www.ethnobiology.net/what-we-do/core-programmes/ise-ethics-programme/code-of-ethics/), and traditional knowledge was carefully collected from various locations in the Kashmir Valley.

**Table 1:** Demographic status of informants from the study area in Kashmir Valley (India)

Villages	Demographic characteristics								
	GPS points		Altitude (m)	Ecology	Ethnicity	Language	Religion	Approx. population	Study participants
Boniyar	34.100251	74.201082	1804	Alpine	Kashmiri Pahari	Kashmiri Pahari	Islam	3454	12
Budwan	34.3872412	73.8914625	2209	Sub-alpine Alpine	Kashmiri Pahari Gujjar	Kashmiri Pahari Gujjar	Islam	4322	11
Bungus	34.3649071	74.0421007	2969	Sub-alpine Alpine	Gujjar	Gujjar	Islam	1500	10
Dardpora	34.497222	74.4125	1709	Sub-alpine Alpine Temperate	Kashmiri	Kashmiri	Islam	3329	11
Darusa	34.622777	74.454722	2743	Sub-alpine Alpine Temperate	Kashmiri	Kashmiri	Islam	2986	11
Gurez	34.6333	74.833	2415	Sub-alpine	Kashmiri	Kashmiri	Islam	29000	12
Naga	34.644916	73.956111	1591	Alpine Temperate	Pahrai	Pahrai	Islam	3750	10
Rajwara	34.40361111	74.25888889	1606	Sub-alpine Alpine Temperate	Kashmiri Pahari	Kashmiri Pahari	Islam	2500	10
Tarbani	34.3808344	73.8244459	1549	Sub-alpine Alpine Temperate	Pahari Gujjar	Pahari Gujjar	Islam	1200	10

To ensure careful examination and the preparation of herbarium specimens, we worked with knowledgeable informants from each study area. For accurate plant identification, we relied on regional literature sources [15, 19–22]. In cases where disagreements over local nomenclature arose, group consensus was reached through intense debate. To achieve accurate taxonomic identification, the collected specimens were examined in detail under the invaluable guidance of taxonomists from Jiwaji University, Gwalior, India. Moreover, the correctness of the nomenclature was confirmed by referring to the WFO (2024) to maintain the highest standards of accuracy and scientific rigor.

### Socioeconomic background

**Fig. 2:**
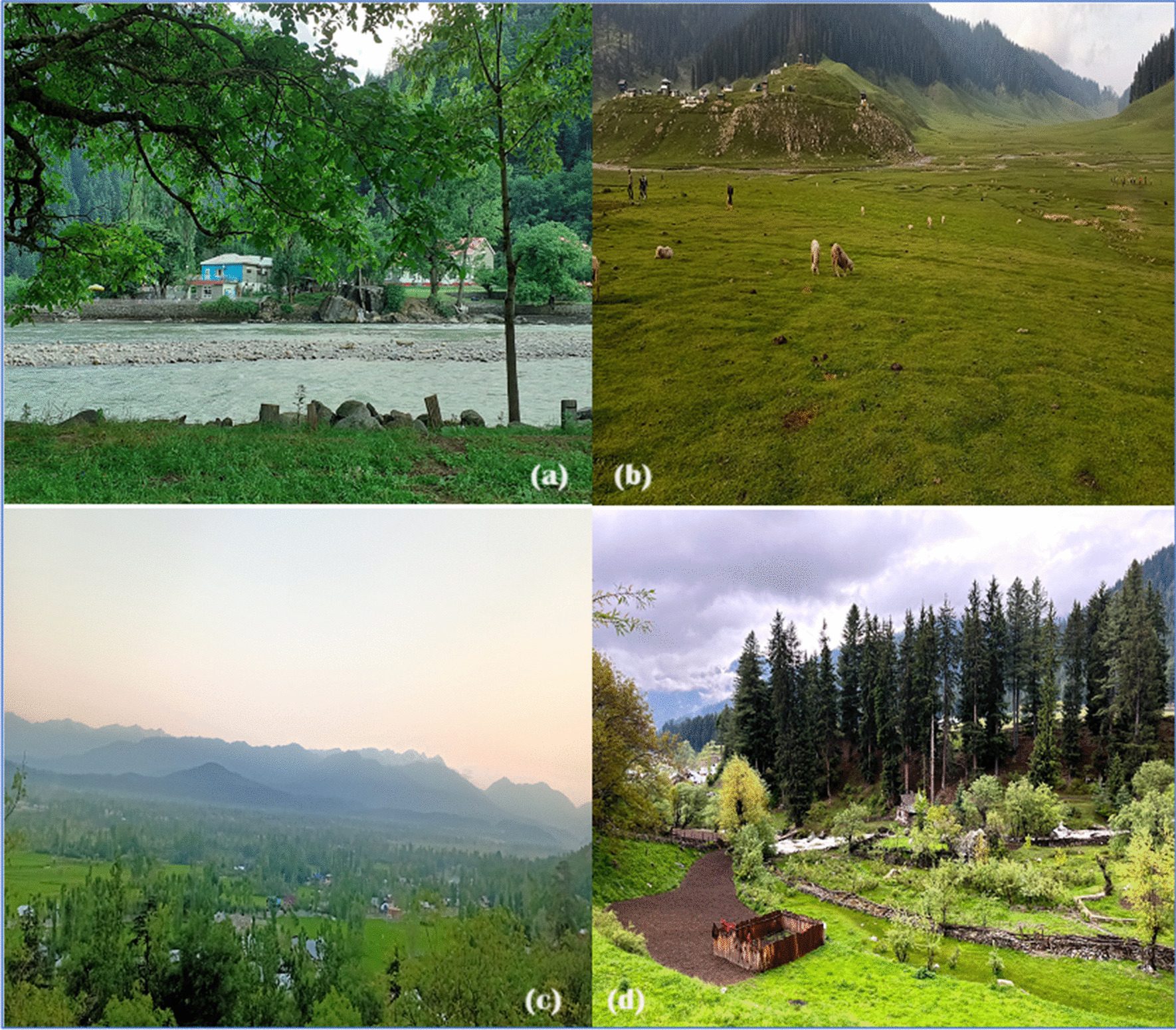
Various villages visited as part of the study in Kashmir Valley (India): **a** Naga; **b**: Bungus; **c**: Tarbani; and **d**: Gurez. (Photograph: Tawseef Ahmad Mir)

The people living in the frontier areas of the Kashmir Valley are particularly dependent on the local ecology, especially through the practice of relying on wild foods [15]. The rugged terrain and often inaccessible landscapes have necessitated a harmonious relationship with nature in which these communities have honed their skills in foraging for wild foods. Wild foods, including a variety of indigenous plants and seasonal produce, contribute significantly to the diet of these frontier communities. This reliance on wild foods not only serves to meet their nutritional needs but is also an expression of a deep connection to the land and its biodiversity. The traditional knowledge in Kashmir, which is passed on from generation to generation, gives them the ability to sustain themselves and shows a remarkable balance between adapting to the environment and preserving cultural heritage in their way of life [23, 24]. Figure 2 shows some of the landscapes examined in the study.

### Data analysis

The use value (UV) is a metric used in ethnobotany to quantify the significance of a plant species based on the number of different uses reported by informants [25]. This measure helps determine a plant’s versatility or utility, reflecting its cultural and practical importance. The utilization value index (UV) was applied to assess the importance of a species to the informants and its gastronomic use among the edible wild species.

Utilization value was calculated using the following formula:

$${\text{UV}} = {\text{Ui}}/{\text{N}}$$

where Ui is the total number of utilization reports of each informant, and N is the total number of informants involved in the study.

Further, a chord diagram was used to show species distribution across the families, and the same was employed to reveal the part usage and specific food use associated with the documented species. Knowledge of the wild food used by the respondents (selected age groups) across the different selected sites in the study area was also represented via a chord diagram, and we used the statistical software Origin Pro 2021. Neighbor-joining clustering was also performed using the PAST software showing the Jaccard similarity index for the different studies. This comprehensive approach enabled a deeper understanding of the data set, revealing the species being reported for the first time, resulting in exploring the novelty of the present study.

## Results and discussion

### Diversity of wild edible plants

**Fig. 3:**
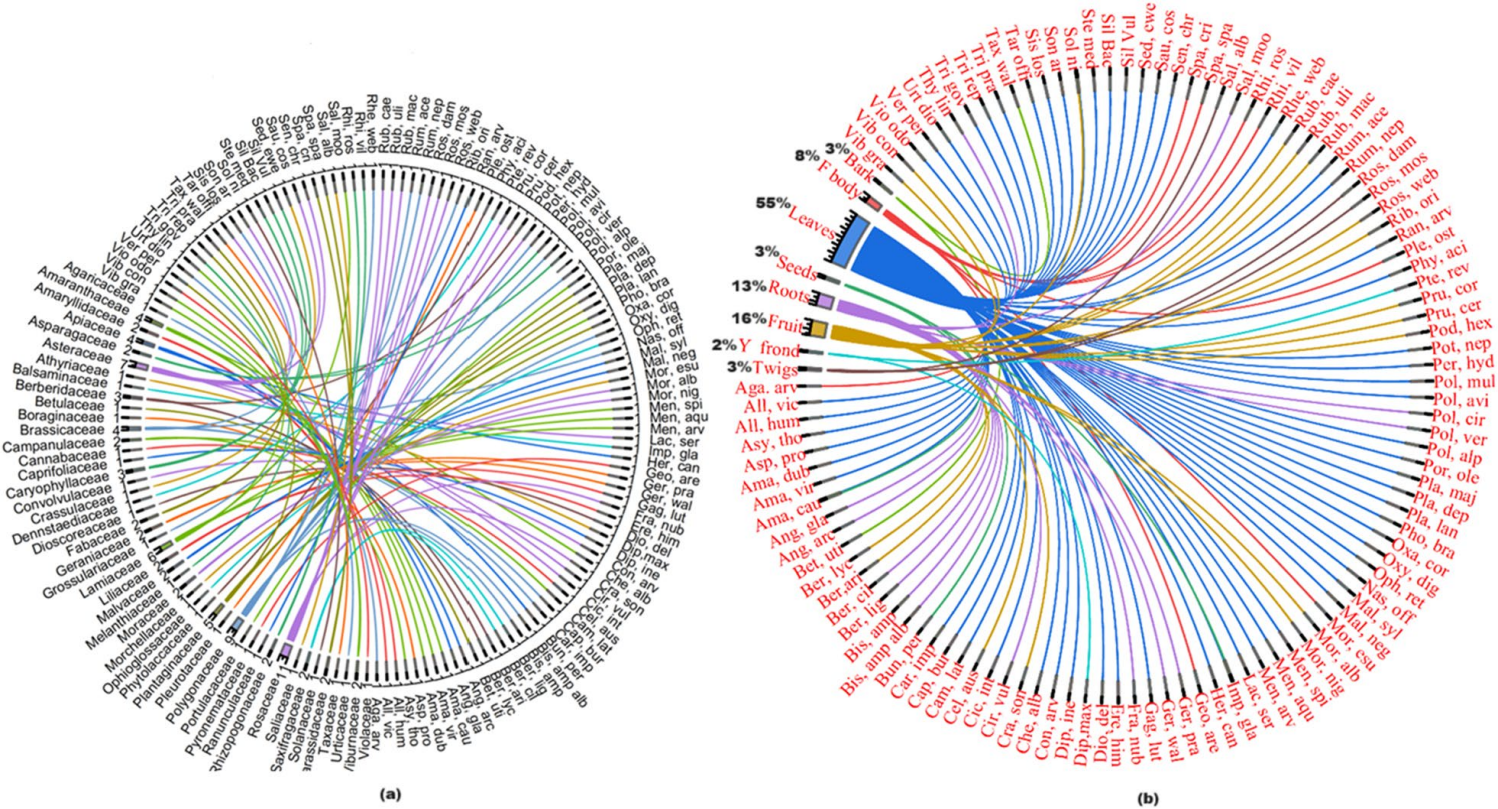
**a** Chord diagram showing the distribution of species across the families; **b** chord diagram showing the percentages of the part usage contributed by the corresponding plant species. The complete names of the species are provided in Table 2

Wild edible plant species are an important source of food for rural populations worldwide. In the current study, a comprehensive inventory of 99 edible plants and 9 fungi species (sum N = 108) from 48 taxonomic families was documented through informant reports (Table 2). The most important family among these was Rosaceae (*N* = 11), closely followed by Polygonaceae (*N* = 9), Asteraceae (*N* = 7), Lamiaceae (*N*= 6), Plantaginaceae (*N* = 5), Amaranthaceae, Apiaceae, and Berberidaceae (*N* = 3 each) (Fig. 3a). The great importance of Rosaceae, Polygonaceae, and Asteraceae is due to the favorable environmental conditions and the suitability of the habitats in the region. In addition, the local population has extensive ecological and traditional knowledge about these families [26–28].

**Table 2:** Inventory of the documented wild edible species used by the people in Kashmir Valley (India)

Botanical name Abbreviation Voucher number	Common name	Life form	Part used	Period of collection	Preparation	Gastronomic use	Medicinal usage	Market food value	UI	UV	IUCN status
*Agaricaceae*											
*Agaricus compestris* L Aga. Com 2968-KASH	Jungle heand	Fungi	Fruiting body	May–August	The fruiting body is fried and cooked	Vegetable	N	×	36	0.37	LC
*Agaricus arvensis* Schaeff Aga. Arv 2967-KASH	Modan heand	Fungi	Fruiting body	May–August	The fruiting body is fried and cooked	Vegetable	N	*×*	28	0.29	LC
*Amaranthaceae*											
*Amaranthus dubius* Mart. ex Thell Ama. Dub 6244-KASH	Krey kul	Herb	Seeds	May–August	Seeds are fried in ghee	Vegetable	Y	*√*	30	0.31	LC
*Amaranthus viridis* L Ama. Vir 3360-KASH	Leeas	Herb	Leaves Seeds	May–August	Seeds and leaves are fried in ghee	Vegetable	Y	*√*	24	0.25	LC
*Amaranthus caudatus* L Ama. Cau 3361-KASH	Leesa	Herb	Leaves Seeds	May–September	Seeds and leaves are fried in ghee	Vegetable	Y	*√*	28	0.29	LC
*Chenopodium album* L Che. Alb 6251-KASH	Leeas	Herb	Leaves	March–June	Leaves are boiled in water and added with cow ghee	Vegetable	Y	*×*	42	0.43	LC
*Amaryllidaceae*											
*Allium victorialis* L All. Vic 3812-KASH	Jungle rohan	Herb	Leaves	April–June	Leaves are fried at low heat	Vegetable	Y	*×*	26	0.27	VU
*Allium humile Kunth* All. Hum 2769-KASH	Jungle pran	Herb	Leaves	May–July	Leaves are fried and kept in the air to make them crispy	Vegetable	Y	*×*	37	0.38	VU
Apiaceae											
*Angelica glauca* Edgew Ang. Gla 4110-KASH	Choor	Herb	Roots	July–August	Dried and used raw	Flavoring agent	Y	*√*	40	0.41	VU
*Angelica archangelica* L Ang. Arc 3336-KASH	Choud	Herb	Roots	July–August	Dried and used raw	Flavoring agent	Y	*√*	38	0.39	VU
*Heracleum candicans* Wall Her. Can 3446-KASH	Mirkul	Herb	Leaves	May–June	Leaves are boiled in water and then added with fried oil	Vegetable	Y	*×*	35	0.36	LC
*Bunium persicum* (Boiss.) B. Fedtsch Bun. Per 2974-KASH	Kala-zeera	Herb	Seeds	June–August	Dried	Flavoring agent	Y	*√*	47	0.48	VU
*Asparagaceae*											
*Polygonatum verticillatum* (L.) All Pol. Ver 4230-KASH	Salamesri	Herb	Roots	June–October	Fresh and raw consumed as salad	Vegetable	Y	*√*	30	0.31	LC
*Polygonatum cirrhifolium* Royle Pol. Cir 4231-KASH	Salapmesri	Herb	Roots	June–October	Fresh and raw consumed as salad	Vegetable	Y	*√*	25	0.26	LC
*Polygonatum multiflorum* (L.) All Pol. Mul 4046-KASH	Watkram	Herb	Leaves	March–May	Leaves are boiled in water and added with boiled oil	Vegetable	N	*×*	26	0.27	LC
*Asteraceae*											
*Cichorium intybus* L Cic. Int 4222-KASH	Haand	Herb	Leaves	May–June	Leaves are added with oil and water and cooked	Vegetable	Y	*√*	55	0.57	LC
*Cirsium vulgaris* (Savi) Ten Cir. Vul 4222-KASH	Kund	Herb	Roots	July–September	Fresh and raw consumed as salad	Vegetable	N	*×*	19	0.20	LC
*Lactuca serriola* L Lac. Ser 2988-KASH	Dudij	Herb	Leaves	March–May	Leaves are boiled in water and added with some ghee	Vegetable	Y	*×*	22	0.23	LC
*Myriactics nepalensis Less* Myr. Nep 3418-KASH	Gahj	Herb	Leaves	July–October	Leaves are boiled in water and added with some ghee	Vegetable	N	*×*	22	0.23	LC
*Cirsium vulgaris* (Savi) Ten Cir. Vul 4222-KASH	Kund	Herb	Roots	July–September	Fresh and raw consumed as salad	Vegetable	N	*×*	19	0.20	LC
*Senecio chrysanthemoides* DC Sen. Chr 4101-KASH	Bough	Herb	Leaves	March–May	Leaves are boiled in water and added with some ghee	Vegetable	Y	*×*	29	0.30	LC
*Saussurea costus* (Falc.) Lipsch Sau. Cos 4211-KASH	Kouth	Herb	Leaves	May–June	Leaves are boiled in water	Vegetable	Y	*√*	31	0.32	CR
*Taraxacum officinale* (L.) Weber ex F.H. Wigg Tar. Off 6259-KASH	Kaw Heand	Herb	Tender leaves	February–April	Leaves are boiled in water and added with some ghee	Vegetable	Y	*√*	57	0.59	LC
*Sonchus arvensis* L Son. Arv 3448-KASH	Dudij	Herb	Tender leaves	March–April	Leaves are boiled in water and are added with some ghee	Vegetable	N	*×*	27	0.28	LC
*Athyriaceae*											
*Diplazium maximum* (D.Don) C.Chr Dip. Max 4001-KASH	Longud	Fern	Young frond	April–May	Leaves are dried, boiled in water, and added with some ghee	Vegetable	Y	*√*	50	0.52	LC
*Balsaminaceae*											
*Impatiens glandulifera* Royle Imp. Gla 2989-KASH	Truil	Herb	Leaves	June–August	Leaves are fried and boiled	Vegetable	Y	*×*	19	0.20	LC
			Seeds		Fresh and raw	Fruit					
*Berberidaceae*											
*Berberis lycium* Royle Ber. Lyc 4102-KASH	Kawdach	Shrub	Fruit	March–July	Raw	Fruit	Y	*×*	42	0.43	LC
*Berberis aristata* DC Ber. Ari 6247-KASH	Chaxmachang	Shrub	Fruit	May–August	Raw	Fruit	Y	*×*	25	0.26	EN
*Podophyllum hexandrum* (Royle) T.S.Ying Pod. Hex 4218-KASH	Wanwagun	Herb	Fruit	June–August	Fresh and raw	Fruit	Y	*×*	47	0.48	EN
*Betulaceae*											
*Betula utilis* D.Don Bet. Uti 4105-KASH	Burz	Tree	Bark	March–September	Bark is boiled in water for more than half an hour	Tea	Y	*×*	29	0.30	EN
*Boraginaceae*											
*Asperugo procumbens* L Asp. Pro 2970-KASH	Bread haakh	Herb	Leaves	May–June	Leaves are fried and added with water and turmeric and kept on low heat for half an hour	Vegetable	N	*×*	19	0.20	LC
*Brassicaceae*											
*Cardamine impatiens* L Car. Imp 2976-KASH	Chead haakh	Herb	Leaves	March–June	Cooked	Vegetable	Y	*×*	16	0.16	LC
*Capsella bursa-pastoris* (L.) Medik Cap. Bur 4250-KASH	Kralmund	Herb	Leaves	March–April	Leaves are fried and added with water and turmeric and kept on low heat for half an hour	Vegetable	Y	*×*	51	0.53	LC
*Nasturtium officinale* W.T.Aiton Nas. Off 4226-KASH	Kul nunnery	Herb	Leaves	March–April	Leaves are fried on low heat	Vegetable	Y	*×*	28	0.29	LC
*Sisymbrium loselli* L Sis. Los 3449-KASH	Dand haakh	Herb	Leaves	April–May	Leaves are boiled and added with turmeric	Vegetable	N	*×*	19	0.20	LC
*Campanulaceae*											
*Asyneuma thomsoni* (C.B.Clarke) Bornm Asy. Tho 2966-KASH	Doodh haakh	Herb	Leaves	March–June	Leaves are fried, added with water, and boiled	Vegetable	N	*×*	15	0.15	LC
*Cannabaceae*											
*Celtis australis* L Cil. Aus 3380-KASH	Brimaj	Tree	Fruit	August–October	Raw	Fruits	Y	*×*	36	0.37	LC
*Caprifoliaceae*											
*Dipsacus inermis* Wall ex Roxb Dip. Ine 4007-KASH	Wapul haakh	Herb	Leaves	May–June	Leaves are dried and boiled for half an hour	Vegetable	Y	*×*	46	0.47	LC
*Caryophyllaceae*											
*Silene vulgaris* (Moench) Garcke Sil. Vul 3441-KASH	Waat kram	Herb	Leaves	April–May	Leaves are boiled in water along with turmeric	Vegetable	Y		34	0.35	LC
*Silene baccifera* Roth*,* Sil. Bac 3445-KASH	Nadam nood	Herb	Leaves	April–May	Leaves are fried and then added with a small amount of water	Vegetable	N	*×*	38	0.39	LC
*Stellaria media (L.)* Vill Ste. Med 4249-KASH	Nick haakh	Herb	Leaves	April–June	Leaves are fried and then added with a small amount of water	Vegetable	Y	*×*	29	0.30	LC
*Convolvulaceae*											
*Convolvulus arvensis* L Con. Arv 3384-KASH	Raiz gass	Herb	Leaves	March–April	Leaves are fried and then added with a small amount of water	Vegetable	Y	*×*	20	0.21	LC
*Crassulaceae*											
*Sedum ewersii* Lebed Sed. Ewe 3003-KASH	Pal nunner	Herb	Leaves	March–May	Leaves are fried and then added with a small amount of water	Vegetable	N	*×*	20	0.21	LC
*Dennstaediaceae*											
*Pteridium revolutum* (Blume) Nakai Pte.rev 7090-KASH	Jungle kunji	Fern	Stem	April–May	Stem is dried and boiled in water along with species like fennel	Vegetable	Y	*√*	42	0.43	LC
*Dioscoreaceae*											
*Dioscorea deltoidea* Wall. ex Kunth Dio. Del 6237-KASH	Shingle-mingle	Climber	Leaves	May–June	Leaves are dried boiled in water, strained, and fried on low heat	Vegetable	Y	*×*	31	0.32	VU
*Fabaceae*											
*Trifolium pratense* L Tri. Pra 3454-KASH	Batakhlout	Herb	Leaves	April–June	Leaves are fired then added water to remove crispiness	Vegetable	N	*×*	25	0.26	LC
*Trifolium repens* L Tri. Rep 3455-KASH	Batakhlout	Herb	Leaves	April–June	Leaves are fired then added water to remove crispiness	Vegetable	N	*×*	32	0.33	LC
*Geraniaceae*											
*Geranium wallichianum* Oliv Ger. Wal 4112-KASH	Ratanjog	Herb	Roots	July–August	Roots are dried and boiled in water for more than half an hour	Tea	Y	*√*	31	0.32	LC
*Geranium pratense* L Ger. Pra 4098-KASH	Ratanjog	Herb	Roots	July–August	Roots are dried and boiled in water for more than half an hour	Tea	Y	*√*	36	0.37	LC
*Grossulariaceae*											
*Ribes orientale* Desf Rib. Ori 4052-KASH	Jangli dash	Shrub	Fruit	June–July	Consumed raw. Also, dried and fried in ghee	Vegetable	Y	*×*	39	0.40	LC
*Lamiaceae*											
*Mentha arvensis* L Men. Arv 4234-KASH	Pudina	Herb	Leaves	March–October	Fresh leaves are grinded mixed with paprika and salt and consumed as a salad	Vegetable	Y	*√*	43	0.44	LC
*Mentha aquatica* L Men. Aqu 4235-KASH	Pudina	Herb	Leaves	March–October	Fresh leaves are grinded and mixed with paprika and salt and consumed as a salad	Vegetable	Y		40	0.41	LC
*Mentha longifolia* L Men. Lon 3415-KASH	Pudine	Herb	Leaves	March–October	Fresh leaves are grinded and mixed with paprika and salt and consumed as a salad	Vegetable	Y	*×*	29	0.30	LC
*Phlomoides bracteosa* (Royle ex Benth.) Kamolin & Makhm Phl. Bra 4066-KASH	Neekantha	Herb	Leaves	June–August	Leaves are boiled in water for more than half an hour	Tea	N	*×*	26	0.27	LC
*Salvia moorcroftiana* Wall. ex Benth Sal. Moo 6256-KASH	Gulkand	Herb	Roots	May–August	Fresh and raw consumed as salad	Vegetable	Y	*×*	30	0.31	LC
*Thymus linearis* Benth Thy. Lin 4107-KASH	Jaind	Shrub	Roots	June–July	Boiled in water for more than half an hour	Tea	Y	*×*	28	0.29	LC
*Liliaceae*											
*Eremurus himalaicus* Desf Ere. Him 4003-KASH	Shel-haakh	Herb	Leaves	April–May	Cooked	Vegetable	Y	*×*	41	0.42	VU
*Gagea lutea* (L.) Ker Gawl Gag. Lut 4002-KASH	Naimiun	Herb	Leaves	February–April	Cooked	Vegetable	N	*×*	21	0.22	LC
*Malvaceae*											
*Malva neglecta* Wallr Mal. Neg 2991-KASH	Sochal	Herb	Leaves	March–April	Leaves are fried	Vegetable	Y	*×*	26	0.27	LC
*Malva sylvestris* L Mal. Syl 2992-KASH	Sochal	Herb	Leaves	May–July	Leaves are fried	Vegetable	Y	*×*	34	0.35	LC
*Melanthiaceae*											
*Trillium govanianum* Wall. ex D.Don Tri. Gov 6230-KASH	Trupattri	Herb	Leaves	April–May	Leaves are fried	Vegetable	Y	*×*	26	0.27	EN
*Moraceae*											
*Morus nigra* L Mor. Nig 3417-KASH	Tul	Tree	Fruit	June–August	Fresh and raw	Fruits	Y	*√*	49	0.51	LC
*Morus alba* L Mor. Alb 3418-KASH	Tul	Tree	Fruit	June–August	Fresh and raw	Fruits	Y	*√*	37	0.38	LC
*Morchellaceae*											
*Morchella esculenta* (L.) Pers Mor. Esu 4215-KASH	Gucchi	Fungi	Fruiting body	March–May	Fruiting body is fried along with onion	Vegetable	Y	*√*	39	0.40	EN
*Ophioglossaceae*											
*Ophioglossum reticulatum* L Oph. Ret 6234-KASH	Chonchur	Fern	Leaves	March–May	Leaves are fried along with shallots	Vegetable	Y	*×*	23	0.24	LC
*Phytolaccaceae*											
*Phytolacca acinosa* Roxb Phy. Aci 4253-KASH	Hapat-chur	Herb	Leaves	April–May	Leaves are fried along with shallots	Vegetable	Y	*×*	33	0.34	LC
*Pinaceae*											
*Abies pindrow* Royle Abi. Pin 2965-KASH	Bunder	Tree	Bark	March–May	Bark is boiled in water for more than half an hour	Tea	Y	*×*	23	0.24	LC
*Plantaginaceae*											
*Oxalis corniculata* L Oxa. Cor 4113-KASH	Chokchrey	Herb	Leaves	March–August	Fresh and Raw consumed as a salad	Vegetable	Y	*√*	32	0.33	LC
*Plantago major* L Pla. Maj 4118-KASH	Bod Gull	Herb	Leaves	March–May	Leaves are fried and boiled in water	Vegetable	Y	*×*	29	0.30	LC
*Plantago lanceolata* L Pla. Lan 6249-KASH	Gull	Herb	Leaves	March–May	Leaves are fried and boiled in water	Vegetable	Y	*×*	40	0.41	LC
*Plantago depressa* Willd Pla. Dep 4067-KASH	Lakut-Gull	Herb	Leaves	April–June	Leaves are fried and boiled in water	Vegetable	N	*×*	35	0.36	LC
*Veronica persica* Poir Ver. Per 3460-KASH	Jungle kalyuth	Herb	Leaves	March–April	Cooked	Vegetable	N	*×*	40	0.41	LC
*Pleurotaceae*											
*Pleurotus ostreatus* (Jacq. ex Fr.) P.Kumm Ple. Ost 2990-KASH	Kul heand	Fungi	Fruiting body	May–October	Fried along with onion	Vegetable	N	*×*	34	0.35	LC
*Polygonaceae*											
*Persicaria amplexicaulis* (D.Don) Ronse Decr Per. Amp 4108-KASH	Manchri chai	Herb	Roots	June–August	Dried roots are boiled in water for more than half an hour	Tea	Y	*×*	43	0.44	LC
*Persicaria nepalensis* (Mesin.) Miyabe Per Nep 6711-KASH	Ratanjosh	Herb	Roots	May–August	Dried roots are boiled in water for more than half an hour	Tea	Y	*×*	39	0.40	LC
*Oxyria digyna* (L) Hill Oxy. Dig 2994-KASH	Chock-abji	Herb	Leaves	May–August	Dried roots are boiled in water for more than half an hour	Vegetable	Y	*×*	28	0.29	LC
*Polygonum alpinum* All Pol. Alp 4041-KASH	Chock chreay	Herb	Leaves	May–July	Fresh and raw consumed as salad	Vegetable	Y	*×*	26	0.27	LC
*Persicaria hydropiper* (L.) Delabre Per. Hyd 3425-KASH	Marchewagan gass	Herb	Leaves	April–May	Fresh leaves are fried	Vegetable	Y	*×*	19	0.20	LC
*Polygonum aviculare* L Pol. Avi 3430-KASH	Druab	Herb	Leaves	March–April	Fresh leaves are fried	Vegetable	Y	*×*	36	0.37	LC
*Rumex nepalensis* Spreng Rum. Nep 6261-KASH	Abij	Herb	Leaves	March–April	Fresh leaves are fried	Vegetable	Y	*×*	46	0.47	LC
*Rumex acetosa* L Rum. Ace 3436-KASH	Aabij	Herb	Leaves	March–April	Fresh leaves are fried	Vegetable	Y	*×*	40	0.41	LC
*Rheum webbianum* Royle Rhe. Web 4212-KASH	Pambchalan	Herb	Leaves	May–June	Fresh leaves are fried	Vegetable	Y	*√*	47	0.48	VU
*Portulacaceae*											
*Portulaca oleracea* L Pro. Ole 3431-KASH	Nunner	Herb	Tender leaves	June–August	Cooked	Vegetable	Y	*×*	59	0.61	LC
*Pyronemataceae*											
*Geopora arenicola* (Lev) Kers Geo. Are 4004-KASH	Shajkan	Fungi	Fruiting body	February–April	Fresh or dried leaves are fried	Vegetable	Y	*√*	45	0.46	LC
*Ranunculaceae*											
*Ranunculus arvensis* L Ran. Arv 3434-KASH	Tull hakh	Herb	Leaves	March–April	Fresh leaves are fried	Vegetable	N	*×*	25	0.26	LC
*Rhizopogonaceae*											
*Rhizopogon villosus* Zeller Rhi. Vil 3437-KASH	Mangde	Fungi	Fruiting body	February–April	The fruiting body is fried with onion	Vegetable	Y	*√*	43	0.44	LC
*Rhizopogon roseolus* (Corda) Th.Fr Rhi. Ros 3438-KASH	Deodar mangde	Fungi	Fruiting body	February–April	The fruiting body is fried with onion	Vegetable	Y	*√*	39	0.40	LC
*Rosaceae*											
*Crataegus songarica* K.Koch Cra. Son 3381-KASH	Ring kul	Tree	Fruit	August–October	Fresh and raw	Fruits	Y	×	34	0.35	LC
*Fragaria nubicola* (Hoof. F) L Fra. Nub 4087-KASH	Ringrish	Herb	Roots	July–August	Fresh roots are boiled in water for more than half an hour	Tea	Y	*×*	53	0.55	LC
					Fruits are eaten raw	Fruit					
*Potentilla nepalensis* Hooker Pot. Nep 3424-KASH	Nun kul	Herb	Leaves	April–May	Fresh leaves are fried	Vegetable	N	*×*	16	0.17	LC
*Prunus cerasifera* Ehrh Pru. Cer 7086-KASH	Aish-auche	Tree	Fruit	July–August	Fresh and raw	Fruit	N	*×*	36	0.37	LC
*Prunus cornuta* (Wall. ex Royle) Steud Pru. Cor 3431-KASH	Chreay fal	Tree	Fruit	July–August	Fresh and raw	Fruit	N	*×*	32	0.33	LC
*Rosa webbiana* Wall. ex Royle Ros. Web 6245-KASH	Jungli poash	Shrub	Fruit	July–August	Dried	Jam	Y	*√*	34	0.35	LC
*Rosa moschata* Herrm Ros. Mos 3433-KASH	Poash	Shrub	Young twigs	March–May	Fresh and raw consumed as salad	Vegetable	Y	*×*	39	0.40	LC
*Rosa damascena* Mill Ros. Dam 3431-KASH	Poash	Shrub	Young twigs	March–May	Fresh and raw consumed as salad	Vegetable	Y	*×*	25	0.26	LC
*Rubus macilentus* Jacquem. ex Cambess Rub. Mac 6254-KASH	Gounch	Shrub	Fruit	June–August	Fresh and raw	Fruits	N	*×*	34	0.35	LC
*Rubus ulmifolius* Schott Rub. Ulm 6252-KASH	But gounch	Shrub	Fruit	June–August	Fresh and raw	Fruits	Y	*×*	38	0.39	LC
*Rubus caesius* L Rub. Cae 6233-KASH	Gounch	Shrub	Fruit	June–August	Fresh and raw	Fruits	Y	*×*	24	0.25	LC
*Saliaceae*											
*Salix alba* L Sal. Alb 3000-KASH	Veer	Tree	Young twigs	March–June	Fresh and raw consumed as salad	Vegetable	Y	*×*	28	0.29	LC
*Saxifragaceae*											
*Bergenia ciliata* (Haw.) Sternb Ber. Cil 4213-KASH	Palfort	Herb	Roots	June–September	Dried roots are boiled in water for more than half an hour	Tea	Y	*√*	38	0.39	VU
*Bergenia ligulata* (Wall.) Engl Ber. Lig 2973-KASH	Palfort	Herb	Roots	August–September	Dried roots are boiled in water for more than half an hour	Tea	Y	*√*	31	0.32	VU
*Solanaceae*											
*Solanum nigrum* L Sol. Nig 3446-KASH	Kambai kul	Herb	Leaves	June–August	Fresh leaves are fried	Vegetable	Y	*×*	46	0.47	LC
			Fruits		Fresh and raw	Fruits					
*Sparassidaceae*											
*Sparassis spathulata* Pk Spa. Spa 3001-KASH	Rai-sair	Fungi	Fruiting body	June–October	Fruiting bodies are fried	Vegetable	N	*×*	36	0.37	LC
*Sparassis crispa* (Wulfen) Fr Spa. Cri 3002-KASH	Rai-sair	Fungi	Fruiting body	June–October	Fruiting bodies are fried	Vegetable	Y	*×*	39	0.40	LC
*Taxaceae*											
*Taxus wallichiana* Zucc Tax. Wal 3450-KASH	Singul	Tree	Bark	April–October	Boiled half an hour minimum	Tea	Y	*×*	29	0.30	EN
*Urticaceae*											
*Urtica dioica* L Urt. Dio 4238-KASH	Soi	Herb	Leaves	March–April	Leaves are fried	Vegetable	Y	*×*	38	0.39	LC
*Viburnaceae*											
*Viburnum continifolium* D.DC Vib. Con 4065-KASH	Kulmanch	Shrub	Fruits	July–October	Fresh and raw	Fruits	Y	*×*	33	0.34	LC
*Viburnum grandiflorum* Wall. ex DC Vib. Gra 4241-KASH	Kilmish	Shrub	Fruit	July–September	Fresh and raw	Fruits	Y	*×*	56	0.58	LC
*Violaceae*											
*Viola odorata* s *(Violaceae)* Vio. Odo 3462-KASH	Banpoash	Herb	Leaves	March–April	Leaves are fried	Vegetable	Y	*×*	19	0.20	LC

Y: with medicinal usage; N: no medicinal usage; × : no market value; market value; √: having market value; IUCN: International Union for Conservation of Nature; LC: least concern; VU: vulnerable; CR: critically endangered; and EN: endangered

Analysis of the results revealed that the most common life form among the documented species was herbs (*n* = 73), followed by shrubs (*n* = 13), fungi, trees (*n* = 9 each), ferns (*n*= 3), and climbers (*N* = 1) (Table 2). These results are consistent with previous studies in the western Himalayas [29, 30]. A comprehensive list of cataloged species can be found in Table 2. The use of these documented species within the ethnic group can be attributed to factors such as plant diversity, accessibility, deep-rooted knowledge of edible wild plant species, healthy condition of forest flora, and economic constraints. A variety of plant parts are used in different culinary preparations, with leaves (55%, *N* = 60), fruits (16%, *N* = 17), roots (13%, *N* = 14), and fruiting bodies (8%, N = 9) being the most commonly used ingredients (Fig. 3b). The predominant use of leaves can be attributed to the fact that they are easy to collect and have a rich phytochemistry [31]. In addition, the leaves are frequently consumed as food in the region [32].

**Fig. 4:**
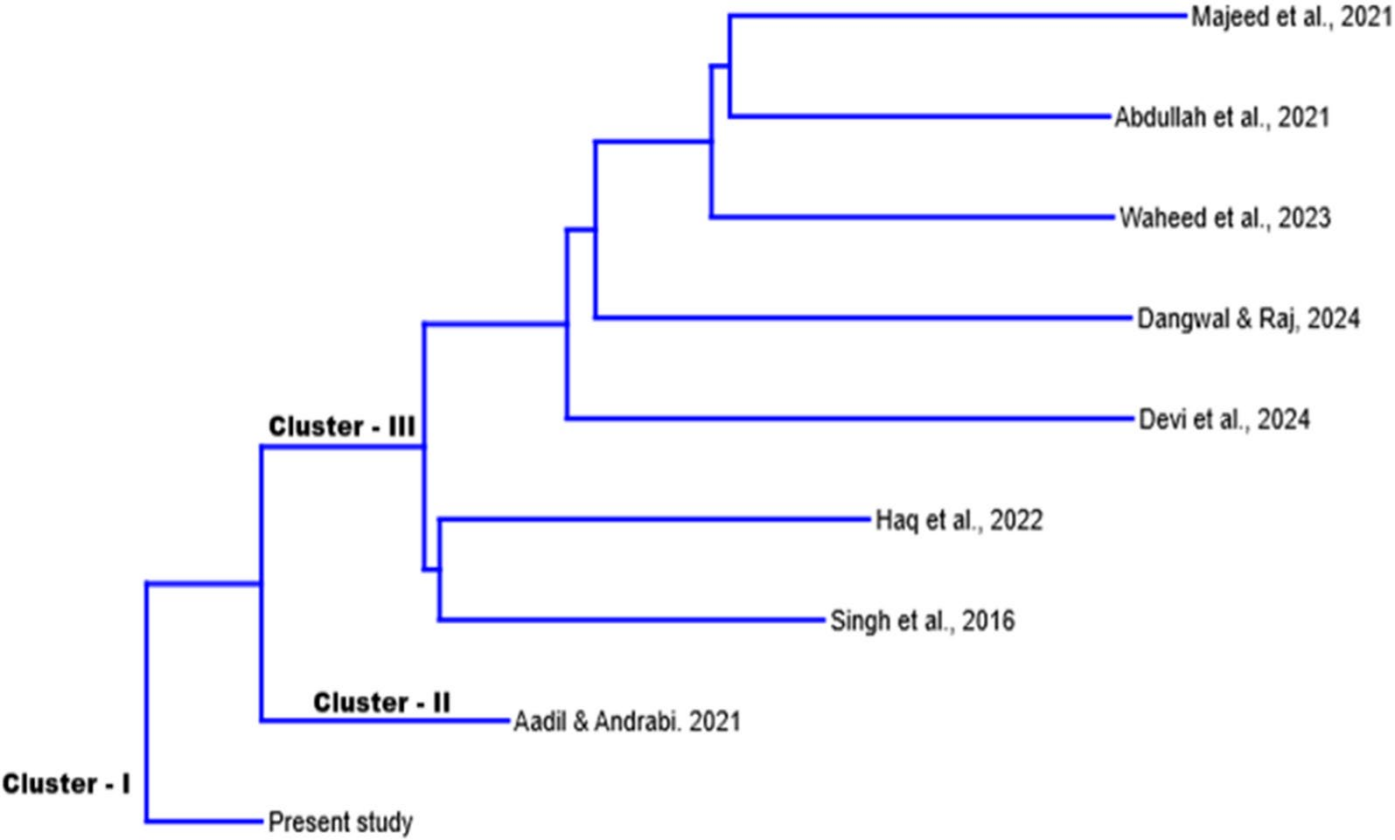
Neighbor-joining clustering displaying the Jaccard similarity index for the different studies across the nearby Himalayan region

Several studies [33–35] have documented the consumption of wild foods in the Kashmir Valley. However, this study is pioneering in reporting the use of wild foods by populations residing near the Line of Control between India and Pakistan. Notably, our study also reported some species previously unreported in this region. To verify and confirm these findings, we compared our results with prior studies [29, 36–41] using Past 4.03 software to plot the Jaccard similarity index via Neighbor-joining clustering (Fig. 4). This index offers profound insights, recognizing both new and gastronomically similar species. Our results revealed 14 species (*Agaricus arvensis, Allium humile, Bergenia ligulata, Campanula latifolia, Cirsium vulgaris, Geranium pratense, Polygonum alpinum, Prunus cerasifera, Pteridium revolutum, Rosa damascene, Rhizopogon villosus, Senecio chrysanthemoides, Sedum ewersii*, and *Sisymbrium loselli*) that had not been previously reported, underscoring the novelty and importance of our study in expanding the understanding of wild food use in the region. Clustering analysis identified three primary clusters (Fig. 4). Cluster I, representing the present study, stands alone, indicating distinct species composition with low similarity and high diversification compared to other studies. This distinctiveness likely results from the unique geographical setting (predominantly mountainous with rich forest cover) and local cultural diversity. Clusters II and III comprise other compared studies, showing varying degrees of similarity, with the cluster farthest from Cluster I being the least similar to the present study.

### Gastronomic use

Despite the widespread reliance on cultivated crops in many societies, people still rely on wild food species [42]. Surprisingly, this ancient practice serves as a food source for at least one billion individuals in their diets. These wild edible plants (WEPs), known for their toughness and adaptability, play a crucial role in addressing important challenges [43]. They contribute to reducing poverty, improving food security, diversifying agriculture, generating income, and combating malnutrition [44]. In simple terms, these plant treasures continue to be highly important in shaping our interconnected ecosystems and overall human well-being.

**Fig. 5:**
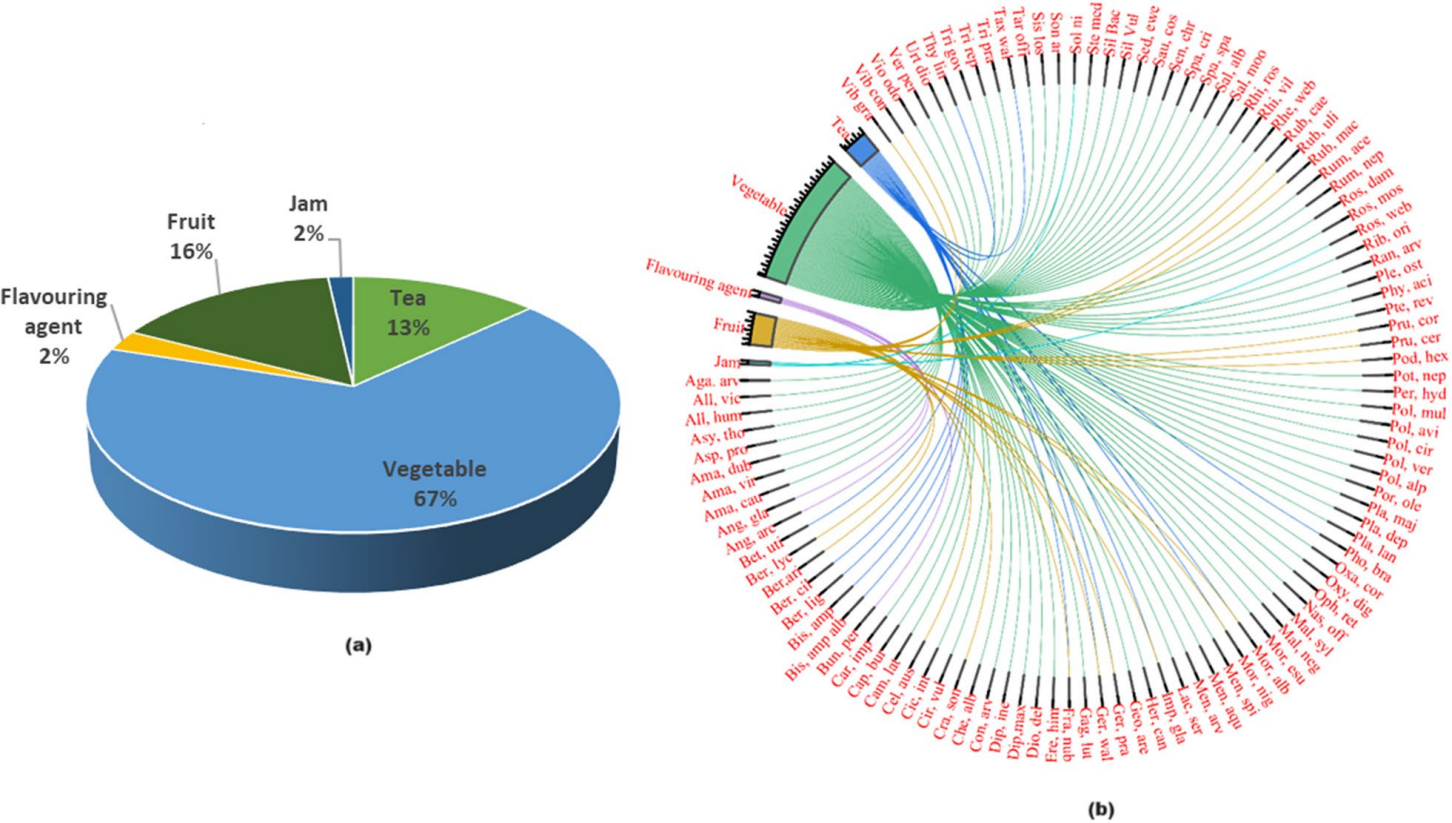
**a** Percentage of different gastronomic uses; **b** Chod diagrams showing the between documented species and their gastronomic use. The complete names of the species are provided in Table 2

In this study, we documented the primary gastronomic uses of various species, i.e., vegetables, fruit, flavoring agents, and tea (Table 2 and Fig. 5a). Among the recorded species, the highest utilization was observed for vegetables (67%, *N* = 77), followed by fruits (16%,*N*  = 18), tea (13%, *N* = 15), flavoring agents (2%, *N* = 3), and jam (2%, *N* = 2). The predominant usage of these species as vegetables can be attributed to traditional practices, limited agricultural land, and inadequate irrigation. Gajural and Doni [44] reported the predominance of vegetable usage upon investing in traditional wild food in the eastern Himalayas. A detailed examination of the data revealed a relatively limited multi-usage pattern among the species. Among all vegetable species, a maximum of 62 were identified with a unique single use. In contrast, the rest of the three species (*Rhizopogon roseolus, R. villosus, and Solanum nigrum*) exhibited dual uses, with *Solanum nigrum* being consumed both as a vegetable and some fruit, and *Rhizopogon roseolus* and *R. villosus* serving as both salad components. Similarly, in the case of tea, 9 taxa (*Abies pindrow, Betula utilis, Bergenia ciliata, B. ligulata, Persicaria amplexicaulis, P. nepalensis****,**** Phlomoides bracteosa, Taxus wallichiana, and Thymus linearis*) were exclusive to tea consumption. At the same time, 6 species (*Morus nigra, M. alba, Impatiens glandulifera, Geranium pratense, Fragaria nubicola, and Geranium wallichianum*) were commonly used for both fruit and tea purposes, demonstrating a dual usage pattern. Similarly, Kunwar et al. [45] reported the single and multistage from west Nepal. Meanwhile, among all documented species, no species displayed shared characteristics across all documented gastronomic attributions. To quantify the relationships between species and gastronomic usage, the chord diagram was employed, providing insights into the gastronomic usage for the corresponding species (Fig. 5b).

The use of herbs as vegetables is widespread throughout the Kashmir Valley. In the present study, we found that women were the primary source of information on the usages of species as vegetables, and most of the women are associated with gastronomic uses, as the kitchen belongs to them culturally in the region and possesses a potential higher knowledge than men. Dad and Khan [46], Pieroni et al. [47]; Singh et al. [35] also reported the knowledge dominance of female folk on gastronomic uses. The main edible parts of the documented species consumed as vegetables include leaves, young fronds, and fruiting bodies (Table 2). *Allium victorialis, Amaranthus dubius, Malva neglecta, Oxalis corniculata, Nasturtium officinale, Oxyria digyna, Cichorium intybus, Plantago depressa, P. major, Rumex nepalensis, Taraxacum officinale, Stellaria media, Silene vulgaris* are the most commonly used species as vegetables. A variety of species (*Mentha longifolia, M. arvensis*, and *M. aquatica*) were also used for making salad which includes specially made dip formally known as *chut/chutney*. Leaves of these species are grounded with a traditional mortar and pestle added with salt, and paprika. The obtained recipe (*chutney*) is mostly consumed fresh. This *chutney* is believed as a potential appetizer also known to treat gastrointestinal disorders if used without paprika. Some fungi species like *Rhizopogon villosus, R. roseolus, Morchella esculenta*, *Geopora arenicola Agaricus campestris, A. arvensis, Pleurotus ostreatus, Sparassis spathulata,* and *S. crispa* are also consumed as vegetables. The usage of fungi is very much praised, even in luxurious weddings the *Morchella esculenta* is served as an important elite dish.

Tea holds a unique role among the different ethnic communities globally [48]. In the present study, tea is locally as known as *Qoda/Cha/Chai*, believed to have warming and revitalizing properties. The consumption of tea tends to surge during the winter season. In our inventory, we documented *N* = 12 plant species used as substitutes for tea. Notably, six species were more frequently employed for this purpose, with only two plant parts, roots and bark, being utilized. These parts are extracted from species such as *Abies pindrow* and *Taxus wallichiana* (bark), *Bergenia ciliata, Persicaria amplexicaulis, Fragaria nubicola, Geranium pratense,* and *Thymus linearis* (roots). The typical preparation method involves boiling the roots or bark in water for an extended duration, often exceeding half an hour, with the addition of salt. Additionally, to intensify the flavor and strength of the tea, it is customary to boil the same plant parts and let them steep overnight, followed by a second round of boiling the next day.

The most used species as fruits are *Berberis lycium, Celtis australis, Rubus caesius, Podophyllum hexandrum, Viburnum grandiflorum, Prunus cerasifera, P. cornuta, Morus nigra*. All the said species are consumed as fresh. There are numerous reports of using wild species as fruits across the globe. Ojelel et al. [49] from Uganda, Mahapatra, and Panda [50], from eastern India, and Khan et al. [51] from Pakistan reported the use of wild species as fruits by the local inhabitants.

The roots of *Angelica glauca* and *A. archangelica* and seeds of *Bunium persicum* were recorded to be employed as flavoring agents. These species are especially added to the local cuisine (*Wazwan*) to enhance the taste. The *Wazwan* a multi-course meal in Kashmiri cuisine, endemic to the region is nowadays practiced in almost all traditional cultures in the valley. It is thought to have originated in Iran and evolved. Spices are the backbone of the *Wazwan*, and traditionally culinary practitioners favor wild species over cultivated or processed species. Our findings agree with Aryal et al. [52] and Bhatia et al. [53], who describe wild plants used in Western Himalayan cuisine, in Udhampur and Jammu, respectively.

### Collection of wild edible food species

**Fig. 6:**
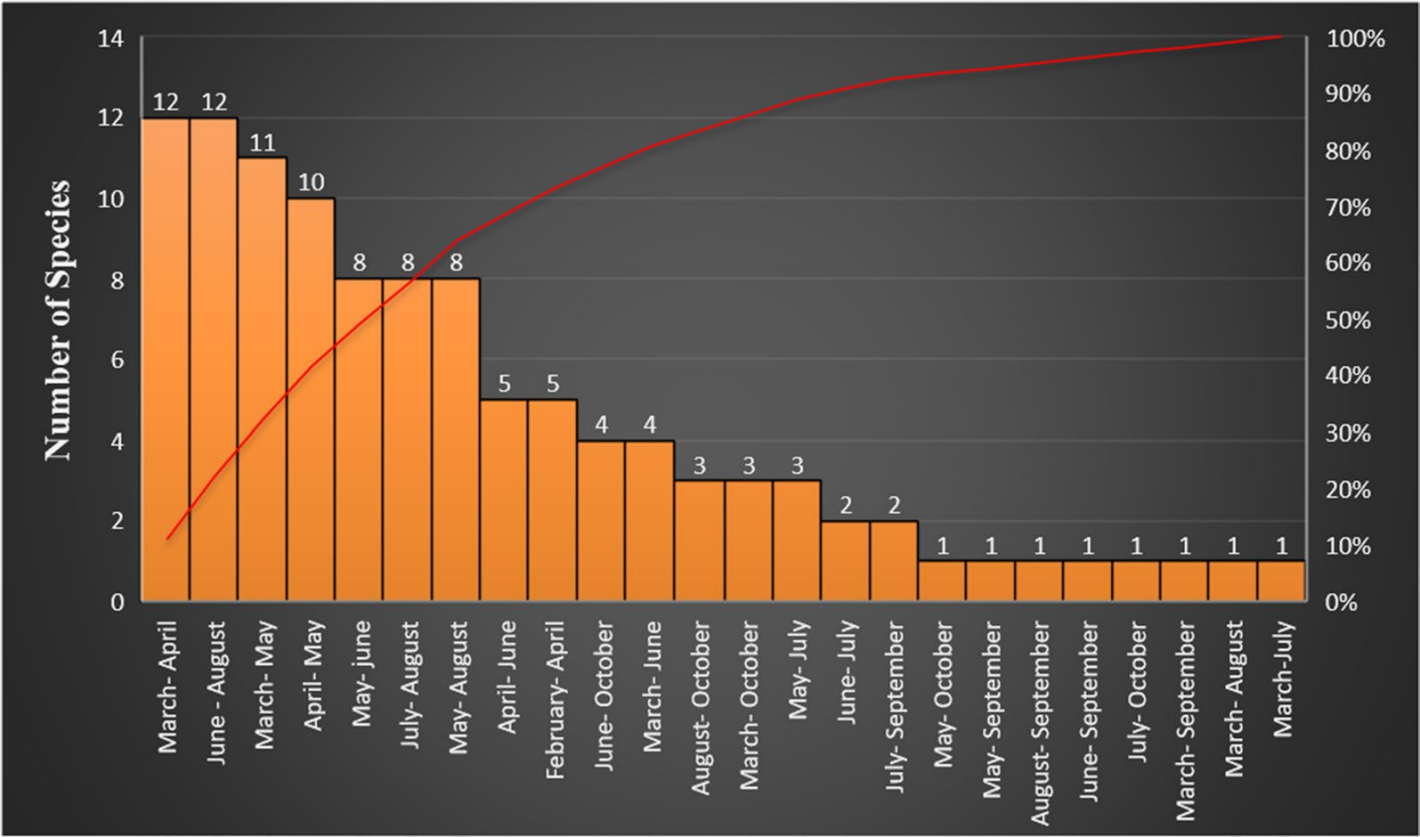
Pareto chart representing the wild edible species collected in the different months in Kashmir

When collecting the listed species, the locals still have a knowledge potential that shows a strong connection to the local flora. Most species (*N* = 12) were collected from March to April, and the same number of species were collected from June to August, followed by *N* = 11 species collected from March to May (Fig. 6). It is important to mention that there is a shortage of cultivated vegetables in the region in March and April due to weather conditions, so people use most wild species in these two months compared to the other months of the year. Species collected in the early spring months include *Capsella bursa-pastoris*, *Convolvulus arvensis, Malva neglecta, Nasturtium officinale, Polygonum aviculare, Ranunculus arvensis, Rumex nepalensis, Rumex acetosa, Sonchus arvensis, Veronica persica, Viola odorata*, and *Urtica dioica*. A complete list can be found in Table 2.

Many species such as *Pteridium revolutum*, *Phytolacca acinosa*, and *Diplazium maximum* are suspected of being poisonous, which is why they are carefully boiled and dried before consumption. These species are also dried in the sun to preserve them for later consumption. The use of wild edible plants (WEP) has some outdated features, such as drying for winter, a tradition that is now only practiced in a few countries around the world [54].

Many species are considered to have medicinal value in addition to gastronomic use (Table 2). It is important to note that the preparations used for cooking are also used as medicine, i.e., species that are consumed as food are themselves medicine. Species such as Portulaca oleracea are used as an immune-boosting agent for postpartum weakness. In addition, the species is also recommended by local traditional healers as a remedy for COVID-19, as it is traditionally considered immune-boosting. Similarly, *Taraxacum officinale, Cichorium intybus,* and *Rheum webbianum* have been used to promote circulation and for general weakness in new mothers. Fruits of *Viburnum grandiflorum* are used for bone and joint ailments.

**Fig. 7:**
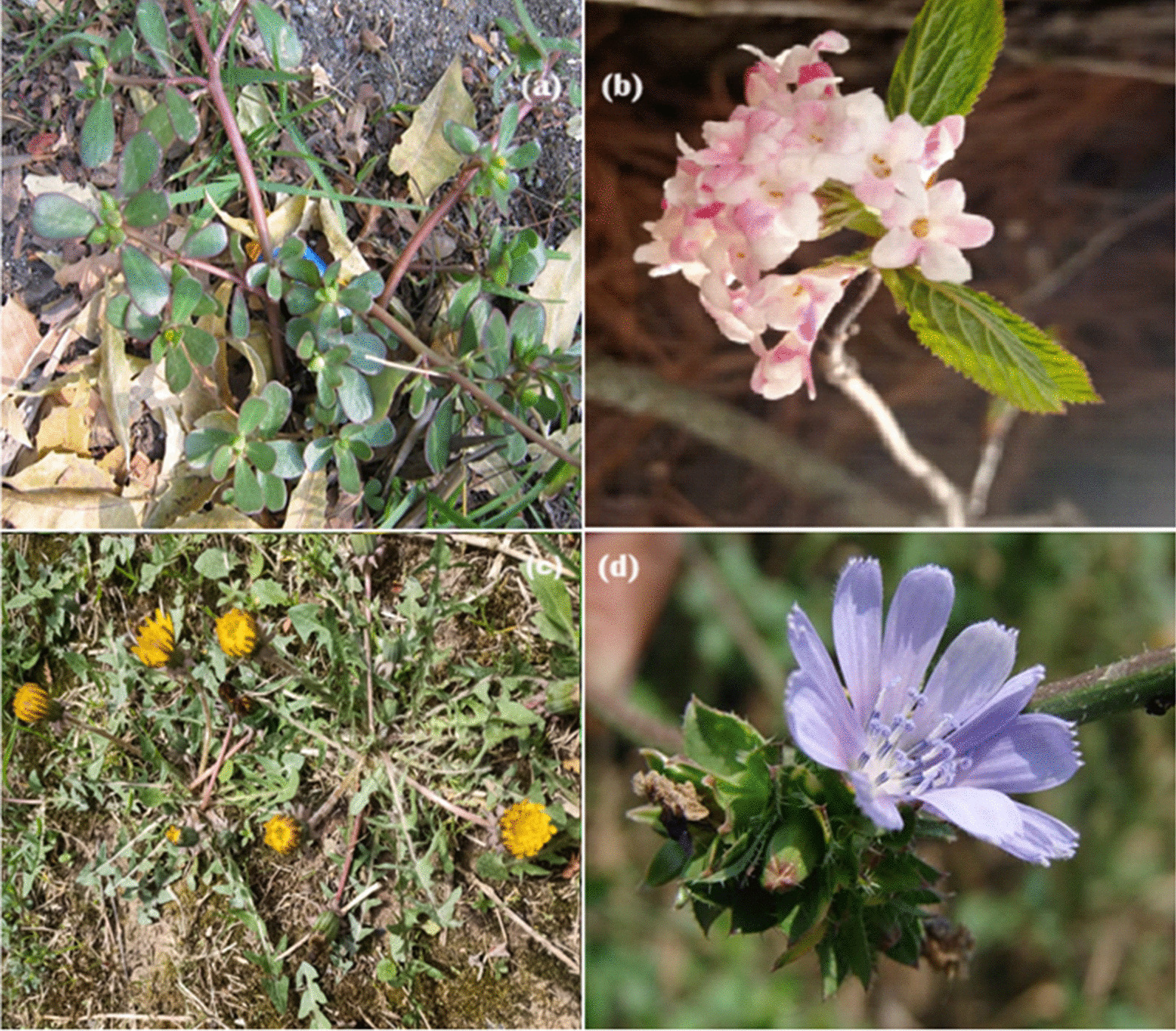
Some of the pictures of wild food species obtained from the study area **a** *Portulaca oleracea*, **b** *Viburnum grandiflorum,* **c** *Taraxacum officinale,* **d** *Viburnum grandiflorum* (Photograph: Tawseef Ahmad Mir)

The use value (UV) indicates the importance of a species for the informants and the gastronomic use of the edible wild species. Based on the UVs, the most popular plant species (Fig. 7) among the inhabitants of the study area were *Portulaca oleracea* (UV = 0.61), followed by *Taraxacum officinale* (UV = 0.59), *Viburnum grandiflorum* (UV = 0.58), *Cichorium intybus* (UV = 0.56) and the lowest use value was reported for *Asyneuma thomsoni* (UV = 0.15) (Table 2).

The highest use value of *Portulaca oleracea* is due to the assumption that the species has a potential nutritional value, which is also supported by the scientific evaluation demonstrating the antioxidant potential and the presence of omega-3 fatty acids [55].

### Cultural importance and food security

**Fig. 8:**
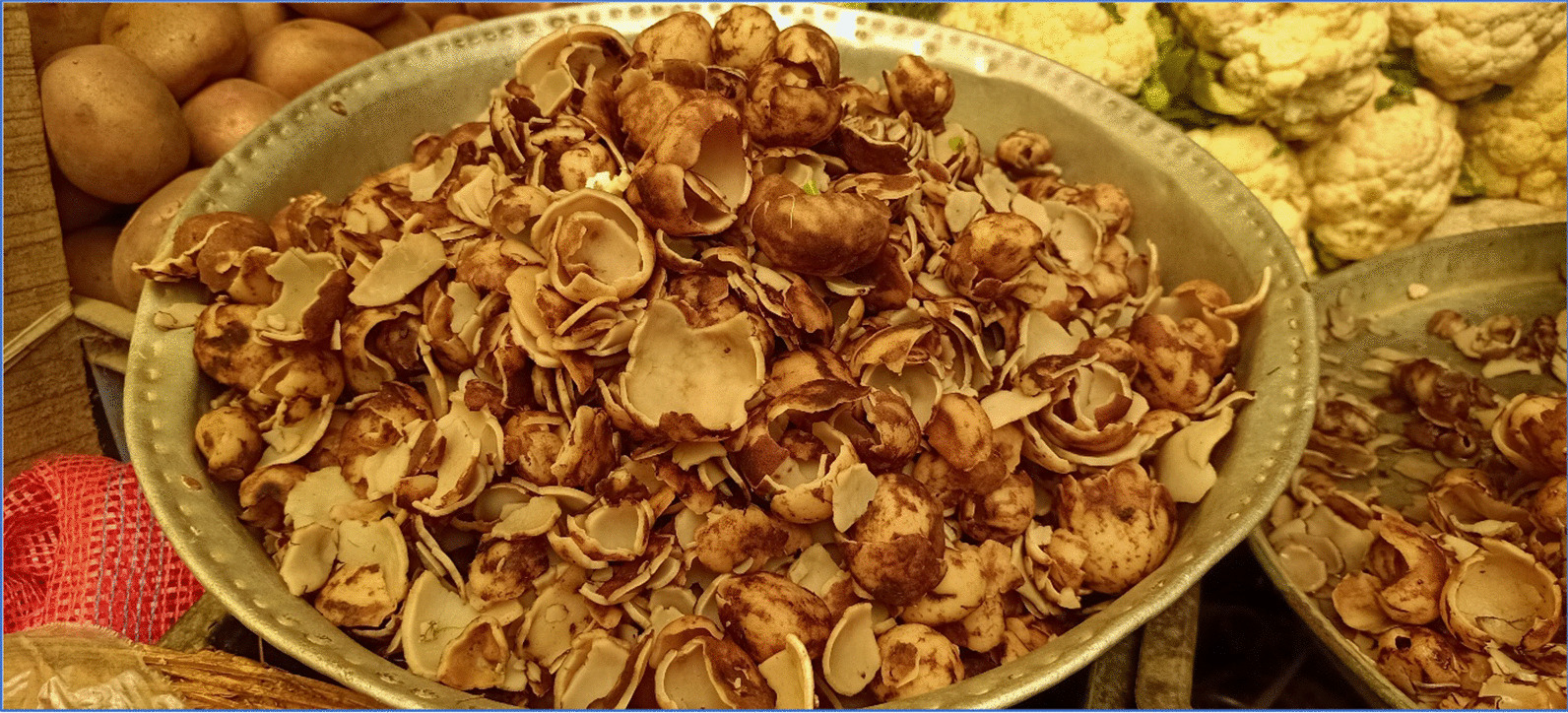
*Geopora arenicola*, a wild edible plant species with market value: (Photograph: Musheerul Hassan)

Throughout the region (Kashmir), several of the documented species have been used in local traditions for centuries [56]. *Betula utilis*, for example, is burnt to produce smoke to ward off evil spirits and is also used by spiritual healers to write scrolls [21]. Similarly, respondents in the present study reported that they keep the part (twig) of *Betula utilis* in the house to avoid bad luck, *Thymus linearis* was used to wash dishes due to its fragrance, and petals of *Rosa webbiana* are dried in the shade and used in tea to enhance aroma and taste. *Geopora arenicola* and *Prunus cornuta* are given to the groom to increase vitality and libido. The ink extracted from *Ribes orientale* is used by local spiritual healers to make amulets. Several documented species, especially various fungi such as *Rhizopogon villosus, R. roseolus, Morchella esculenta,* and *Geopora arenicola* (Fig. 8), play a central role in shaping the social hierarchy within the community. These fungi are highly prized, and their consumption is often associated with elite gatherings such as weddings, celebrations, and feasts that symbolize wealth. *Morchella esculenta* is a prime example of such a species. Other species such as *Geopora arenicola* and *Agaricus compestris* also share a similar fate and contribute significantly to social mobility. It is noteworthy that the locals’ belief in the medicinal properties of these wild food species, in addition to their culinary value, serves as a motivating factor for their continued popularity and consumption.

The study area experiences severe winter conditions, particularly between December and February, resulting in food shortages, notably of vegetables, due to heavy snowfall. Consequently, the local population faces inflated prices for available food items, disproportionately affecting those living below the poverty line. However, the region’s abundance of wild food species provides a vital alternative to cultivated crops. Species like *Rhizopogon villosus, R. roseolus, Morchella esculenta, Agaricus compestris,* and *Geopora arenicola* are readily accessible in markets, with many individuals selling them from their homes. Additionally, species such as *Diplazium maximum, Pteridium revolutum, Geranium wallichianum, Rheum webbianum*, and *Bergenia ciliata* hold economic significance.

The promotion of these wild food plants in the region offers a promising avenue for advancing food sovereignty and ecological sustainability. Embracing the principles of food sovereignty, which advocate for community control over food systems, locals can revive traditional practices associated with wild food species. This includes incorporating these species into local diets to diversify food sources, reduce reliance on conventional agriculture, and market (which is affected by winter), and enhance self-sufficiency and resilience.

Furthermore, the utilization of wild species holds substantial potential for bolstering food security in the region. Practices like “wildlife stewardship,” encompassing traditional land management, seed conservation, and agroforestry systems, can facilitate this endeavor. Policymakers at both state and central levels must enact supportive policies to realize this potential. Additionally, engaging the younger generation through educational initiatives in schools and communities can instill pride in traditional heritage and impart knowledge about the cultural significance of local species. Community events and festivals highlighting the traditional use of these species can further encourage youth participation. Hands-on learning experiences, such as field trips led by local experts, offer direct interaction with the environment and deepen understanding of the cultural importance of local species.

### Intergenerational transfer of traditional wisdom

**Fig. 9:**
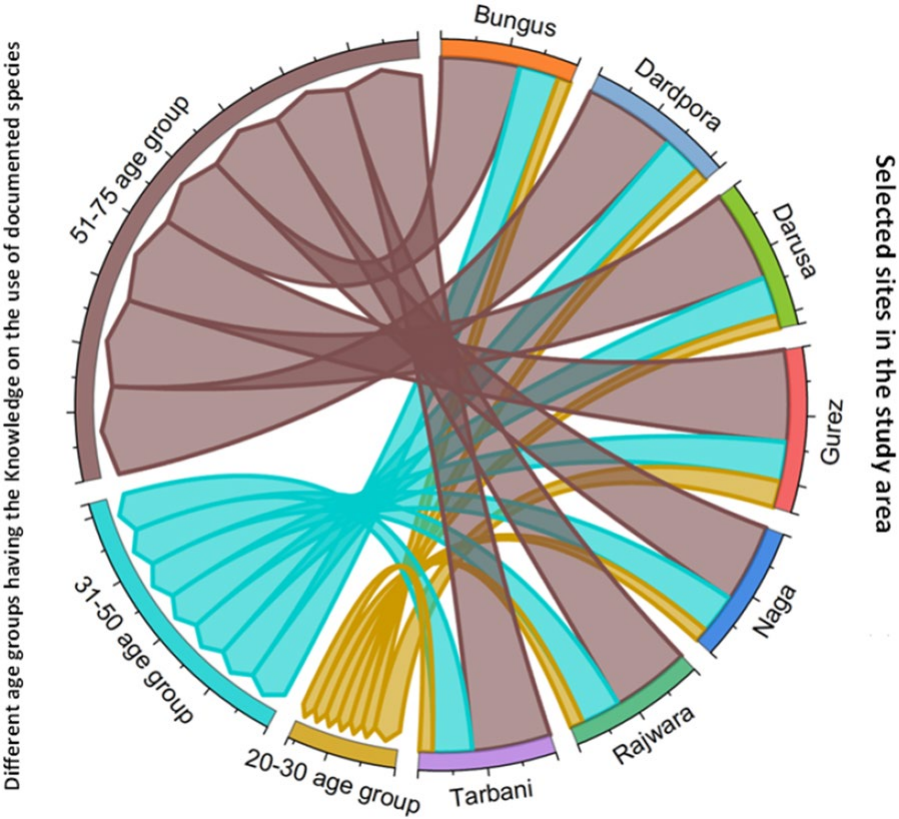
Chord diagram showing the number of respondents from selected age groups having gastronomic knowledge for the documented species in Kashmir Valley (India)

The transmission of knowledge from one generation to the next is crucial for the preservation of cultural heritage, the conservation of biodiversity, and the maintenance of the connection between communities and their local environment [57]. However, our results suggest that traditional knowledge of local wild food resources is no longer passed between generations, as Fig. 9 shows. Our results are in line with [58, 59]. Respondents in our study were selected using the snowball method, where knowledgeable individuals were selected to participate. We found that older individuals were more knowledgeable compared to the younger generation, which they acknowledged. They also gave various reasons for this change. Several factors contribute to the changing knowledge landscape, including cultural changes, urbanization, shifts in family dynamics, and the lure of modern life, technology, and convenience. The media and capitalist influences have also helped to shape the views and behaviors of younger generations. At the same time, modern education is changing people’s attitudes toward modern lifestyles, which in turn is prompting the younger generation to change their lifestyles and move to urban areas, especially the capital city (Srinagar). The studies of Dweba and Mearns [60] and Hanazaki [61] are along the same lines.

To ensure the survival of local plant knowledge, it is important to carry out community programs, cultural initiatives, documentation projects, and research efforts [62]. These initiatives should focus on the preservation of traditional knowledge while considering the changes in culture and society.

Community programs: Engaging local communities in educational programs and workshops can foster a sense of ownership and pride in one’s plant knowledge. These programs can include activities such as plant identification walks, traditional medicine workshops, and gardening initiatives that encourage hands-on learning and participation.

*Cultural initiatives* Incorporating traditional practices and ceremonies into educational initiatives can help to reinforce the cultural importance of local plant knowledge. This could include events such as seed exchange festivals, storytelling about the importance of plants in local folklore, and celebrations of traditional harvesting rituals.

*Documentation projects* Documenting local plant knowledge through interviews with community elders, oral traditions, and written records ensures that valuable information is preserved for future generations. This documentation can take various forms, such as written texts, audio recordings, videos, and digital databases so that the information is accessible and can be easily shared.

*Research efforts* Scientific research into local plants, their uses, and their ecological significance deepens our understanding and appreciation of traditional plant knowledge. Collaborative research projects involving both local communities and academic institutions can provide valuable insights into the medicinal properties, ecological functions, and cultural significance of local flora. While the preservation of traditional knowledge is crucial, it is important to recognize that cultures are dynamic and constantly evolving. Initiatives should be flexible and inclusive, allowing for the incorporation of new perspectives and practices while respecting and honoring traditional forms of knowledge.

### Conservation of wild edible food species

In the present study, we found that the conservation of certain plant species such as *Trillium govanianum, Taxus wallichiana, Saussurea costus, Podophyllum hexandrum, Dioscorea deltoidea, Bunium persicum, Berberis aristata, Betula utilis, Angelica glauca*, and *Allium victorialis* is of utmost importance given their inclusion in the IUCN Red List (Table 2). Consideration of the conservation needs of these species must take precedence over the implementation of proactive mitigation measures. A prevailing consensus in the current research literature from different regions of the world, reflected in studies such as Jiri et al. [63] and Kang et al. [64], emphasizes sociocultural factors as the predominant drivers of dwindling use of wild edible plant species (WEPs). In the present study, we found that a large majority of respondents (*n* = 61) discussed the declining availability of edible wild plants (WEPs) in the Anthropocene. This trend is mainly attributed to the continuous exploitation of certain plant species, primarily due to urbanization, traditional medicinal practices, and economic incentives.

In today’s globalized world, road networks play a crucial role in connecting communities and facilitating economic and social interactions. However, the construction of roads has significant negative impacts on natural ecosystems, including physical disturbance and habitat fragmentation. This often leads to biodiversity loss and habitat degradation, as observed in our study and consistent with the findings of Strittholt and Dellasala [65]. In addition, the increased demand for certain plant species (e.g., *Podophyllum hexandrum*) on the market exacerbates the exploitation of natural resources by local communities for economic reasons [66]. Many plant species are harvested for their traditional medicinal properties. *Taxus wallichiana*, known as Himalayan yew, for example, is highly sought after for its anticancer properties, leading to excessive harvesting. *Trillium govanianum*, valued for its traditional medicinal use to treat sexual disorders, is also at risk of being over-harvested for its alleged therapeutic effects. In addition, species such as *Dioscorea deltoidea*, known for its role in regulating male and female sex hormones, are endangered by unsustainable harvesting practices and pose an imminent threat to their populations and the ecosystems they inhabit. In this context, sustainable management is essential for the conservation of biodiversity and the preservation of ecosystem integrity. Effective strategies include rigorous assessment and monitoring of plant populations (through a combination of field surveys by competent authorities), habitat conservation measures, and enforcement of legislation to prevent overexploitation. In addition, community engagement, educational initiatives, public awareness, and collaboration between stakeholders are crucial to promote responsible harvesting practices and ensure the long-term sustainability of wild plant resources [42].

## Conclusions

Considering the prevailing global reliance on cultivated crops, the continued utilization of wild edible species remains indispensable for sustaining the nutritional needs of a substantial portion of the global population, exceeding one billion individuals. These species serve diverse functions encompassing poverty alleviation, bolstering food security, fostering agricultural diversification, generating economic avenues, and ameliorating malnutrition, with far-reaching implications extending to ecological interdependencies. The present investigation delineates the pivotal role of wild food species in the dietary practices of communities inhabiting the border regions of the Kashmir Valley. Constrained by limited agricultural expanses and logistical constraints in transportation, wild food sources assume a fundamental role as dietary staples. Notably, the seasonal collection of species is a prominent practice, with select taxa, such as *Morchella esculenta* and *Geopora arenicola*, significantly influencing social hierarchies within local communities. Nevertheless, the continuity of traditional knowledge transmission across successive generations confronts discernible challenges, primarily attributed to the disruptive impacts of urbanization on lifestyle trajectories. Urgent conservation strategies are imperative, particularly for endangered plant species cataloged in the IUCN Red List, exemplified by entities like *Trillium govanianum* and *Taxus wallichiana*. Proactive interventions are indispensable to safeguard biodiversity and the associated reservoirs of traditional knowledge. Concurrently, research investigations into the nutritional attributes of wild species stand poised to underpin commercial cultivation endeavors, thus engendering economic prosperity within local spheres and fostering advancements in scientific comprehension. Moreover, the strategic prioritization of conservation initiatives for imperiled species, advocacy for sustainable harvesting methodologies, ethnobiological documentation of Indigenous knowledge systems, and dissemination of awareness among pertinent stakeholders are essential. Educational initiatives and community outreach initiatives emerge as pivotal mechanisms for engendering a paradigm of sustainable utilization and conservation of wild edible flora.

## Data Availability

All the required data are provided in the article.

## Abbreviations

TK: Traditional knowledge
LoC: Line of control
UV: Utilization value index
Ui: Total number of utilization reports of each informant
N: The total number of informants involved in the study
IUCN: International Union for Conservation of Nature
LC: Least concern
VU: Vulnerable
CR: Critically endangered
EN: Endangered
WEPs: Wild edible plants
